# Supplementary material for: A feasibility study evaluating the uptake, effectiveness and acceptability of routine screening of pregnant migrants for latent tuberculosis infection in antenatal care: a research protocol
Source: BMJ Open. 2022 Apr 3;12(4):e058734. doi: 10.1136/bmjopen-2021-058734 (PMC8981348; doi:10.1136/bmjopen-2021-058734)
Supplement: Author's manuscript [file bmjopen-2021-058734.draft_revisions.pdf]

BMJ Open

BMJ Open is committed to open peer review. As part of this commitment we make the peer review history of every article we publish publicly available.

When an article is published we post the peer reviewers' comments and the authors' responses online. We also post the versions of the paper that were used during peer review. These are the versions that the peer review comments apply to.

The versions of the paper that follow are the versions that were submitted during the peer review process. They are not the versions of record or the final published versions. They should not be cited or distributed as the published version of this manuscript.

BMJ Open is an open access journal and the full, final, typeset and author-corrected version of record of the manuscript is available on our site with no access controls, subscription charges or pay-per-view fees (http://bmjopen.bmj.com).

If you have any questions on BMJ Open's open peer review process please email info.bmjopen@bmj.com

BMJ Open

A feasibility study evaluating the uptake, effectiveness and acceptability of routine screening of pregnant migrants for latent tuberculosis infection in antenatal care: a research protocol

Journal:	*BMJ Open*
Manuscript ID	bmjopen-2021-058734
Article Type:	Protocol
Date Submitted by the Author:	26-Oct-2021
Complete List of Authors:	Rahman, A; Queen Mary University of London<br>Zenner, D; Queen Mary University of London, Institute of Population Health Sciences<br>Thangaratinam, Shakila; Queen Mary University of London, BARC (Barts Research Centre for Women's Health), Blizard Institute, Barts and the London School of Medicine and Dentistry<br>Copas, Andrew; UCL<br>White, Peter; Imperial College, MRC Centre for Global Infectious Disease Analysis and NIHR Health Protection Research Unit in Modelling and Health Economics; Public Health England, Modelling and Economics Unit<br>Griffiths, Chris; Barts and The London School of Medicine and Dentistry,<br>Abubakar, Ibrahim; Health Protection Agency, Tuberculosis Section, Centre for Infections<br>McCourt, Christine; Department of Midwifery and Child health, City University London<br>Kunst, Heinke; Queen Mary University of London, Department of Respiratory Medicine
Keywords:	Antenatal < GENETICS, Tuberculosis < INFECTIOUS DISEASES, Public health < INFECTIOUS DISEASES

SCHOLARONE™
Manuscripts

A feasibility study evaluating the uptake, effectiveness and acceptability of routine screening of pregnant migrants for latent tuberculosis infection in antenatal care: a research protocol

Rahman A[1], Thangaratinam S[2], Copas A[3], Zenner D[4], White PJ[5,6], Griffiths C[4], Abubaker I[3], McCourt C[7], Kunst H[1]

[1]Blizard Institute, Barts and The London School of Medicine and Dentistry, Queen Mary University of London, London E1 2AT
[2] WHO Collaborating Centre for Women's Health, Institute of Translational Medicine, Heritage Building, Birmingham, B15 2TH
[3] Institute for Global Health, University College London, Mortimer Market Centre, London, WC1E 6JB
[4] Institute of Population Health Sciences, Queen Mary University of London, London E1 2AB
[5] MRC Centre for Global Infectious Disease Analysis and NIHR Health Protection Research Unit in Modelling and Health Economics, School of Public Health, Imperial College London, Norfolk Place, London, W2 1PG, UK
[6] Modelling and Economics Unit, National Infection Service, Public Health England, London, NW9 5EQ, UK
[7] Centre for Maternal & Child Health Research, City, University of London

Corresponding Author
Dr Ananna Rahman,
Blizard Institute,
Barts and The London School of Medicine and Dentistry, Queen Mary University,
4 Newark Street, London E1 2AT, UK.
E-mail: ananna.rahman1@nhs.net

For peer review only

Abstract

Introduction

Globally, Tuberculosis (TB) is a leading cause of death in women of reproductive age and there is high risk of reactivation of latent tuberculosis infection (LTBI) in pregnancy. The uptake of routine screening of migrants for LTBI in the UK in primary care is low. Antenatal care is a novel setting which could improve uptake and can lend insight into the feasibility and acceptability of offering opt-out screening for LTBI.

Methods and analysis

This is an observational feasibility study with a nested qualitative component. The setting will be the antenatal clinics in three hospitals of an NHS Trust. Inclusion criteria are pregnant migrant women aged 16-35 years attending antenatal clinics who are from countries with a TB incidence of greater than 150/100,000 including sub-Saharan Africa, and who have been in the UK for less than 5 years. Participants will be offered LTBI screening with an opt-out interferon gamma release assay (IGRA) blood test, and be invited to complete a questionnaire. Both participants and healthcare providers will be invited to participate in semi-structured interviews or focus groups to evaluate understanding, feasibility and acceptability of routine opt-out LTBI screening. The primary analysis will focus on estimating the uptake of the screening programme along with the corresponding 95% confidence interval. Secondary analysis will focus on estimating the test positivity. Qualitative analysis will evaluate the acceptability of offering routine opt-out LTBI screening to participants and healthcare providers.

Ethics and dissemination

The study has received the following approvals: Health Research Authority (IRAS 247388) and National Health Service Ethics Committee (19/LO/0557). The results will be made available locally to antenatal clinics and primary care physicians, nationally to NHS England and Public Health England and internationally through conferences and journals.

Trial Registration Number: NCT04098341, pre-results.

Article Summary

Strengths and limitations of this study:

- This is the first study to investigate the uptake, feasibility and acceptability of routine opt-out screening for LTBI in antenatal care in the UK.
- The results will inform ways to increase uptake of LTBI screening in migrants in other settings such as primary care
- A primary limitation of this study is the difficulty in recruiting women who decline LTBI screening to semi-structured interviews and focus group, thereby losing valuable perspectives.

For peer review only - http://bmjopen.bmj.com/site/about/guidelines.xhtml

- Based on these results we will develop a definitive large-scale cluster randomized controlled trial to evaluate the effectiveness of LTBI screening in antenatal care.

Introduction

Context

Tuberculosis (TB) remains a significant global health problem affecting an estimated 10 million people worldwide in 2019 leading to 1.4 million deaths (1). TB is one of the top 10 causes of death globally and the leading cause from any single infectious agent (more than HIV/AIDS) (1) . A quarter of the world's population is estimated to have latent TB infection (LTBI) (2)The World Health Organisation defines LTBI as a 'state of persistent immune response stimulation by *Mycobacterium Tuberculosis* antigens without evidence of clinically manifested active TB' (1). Individuals with LTBI have no signs and symptoms of active TB but remain at risk of developing active TB in their lifetime. LTBI acts as a reservoir for active TB and TB elimination requires strategies for LTBI control (3). Uptake of LTBI screening in primary care is low (4, 5). Antenatal care is a new setting for LTBI screening and understanding the factors affecting the feasibility and acceptability of LTBI screening in this setting are first steps towards developing effective interventions to improving LTBI screening uptake.

Current knowledge

In women of childbearing age (16-45years), TB is one of three leading causes of death globally (2). Diagnosis of TB in pregnancy is often delayed as pregnancy can mask some of the clinical manifestations of TB (6, 7). There is a higher risk of LTBI reactivation during pregnancy and postpartum likely due to T-cell suppression and reduced interferon-gamma production (8). TB in pregnancy is associated with poor perinatal, foetal and maternal outcomes (9, 10).

The UK has one of the highest TB incidence rates in Western Europe. The incidence of TB among those born outside the UK is 14 times higher at 39.0 per 100,000 population and accounting for 74% of all new cases of TB in England in 2019 (11). Public Health England's TB migrant health guide strategy recommends migrant screening for LTBI in high incidence areas in England such as the London Boroughs of Tower Hamlets, Newham and Waltham Forrest (12, 13).

The London Borough of Newham was spearheading large-scale LTBI screening programme in anticipation of the national programme. A total of 20,905 LTBI tests were reported between July 2014 and June 2017 across England with nearly half of the tests taking place in Newham (4, 5). Between April 2015 and June 2016, 5,622 eligible migrants in England were offered an LTBI test, 2,904 (51%) of whom attended for the test (4).

For peer review only

Effective screening for LTBI is key to reducing TB incidence in the UK. There is good evidence that screening and treatment of LTBI is a cost-effective intervention that significantly reduces the risk of developing active disease and the risk of onward transmission (14, 15). The national LTBI migrant screening programme has been rolled out but there is insufficient evidence on the best setting for uptake of LTBI screening.

Rationale for LTBI screening in antenatal care

Pregnancy can predispose to reactivation of LTBI and diagnosis can be delayed due to reduced awareness among healthcare providers and reluctance to investigate non-specific TB symptoms by chest radiography (16). Risks of LTBI reactivation and delays in diagnosis of TB can be mitigated by screening an at-risk pregnant migrant population for LTBI. A simple clinical algorithm recommended by the WHO based on absence of current cough, fever, weight loss, and night sweats can help to exclude active TB disease. Moreover, healthcare professionals will have a higher index of suspicion for active TB in IGRA positive pregnant migrant women presenting with symptoms suggestive of TB, thus preventing a delay in diagnosis (17).

Pregnant migrants may not be accessing routine health care and often do not have a GP. Antenatal care may therefore be a key opportunity to assess the woman's health and screen for TB. Antenatal care provides an opportunity for health promotion such as advocating GP registration and is a time when parents may be particularly receptive to public health information and promotion.

LTBI screening for migrants from high TB incidence countries in antenatal care has shown high uptake in the U.S. but feasibility of LTBI screening in antenatal clinics in the UK has not been evaluated (18).

Research hypothesis and aims

We hypothesise that offering routine opt-out LTBI screening to an at-risk pregnant migrant population in antenatal care will be feasible and acceptable to pregnant migrant women and healthcare providers.

There is limited qualitative research about the acceptability to women of LTBI screening in pregnancy. Reasons for low uptake may be due to stigma of having active TB or fear of a positive test result affecting their immigration status. An opt-out approach to LTBI screening may normalise the process and has the potential to reduce barriers such as stigma, as well as practical barriers (19).

Provider knowledge and understanding of the risks of TB, screening and treatment can be a major predictor of successful management of TB (20). Data from Newham's LTBI screening programme has highlighted that offer of screening varies amongst GP practices indicating that health care provider knowledge and attitude may influence offer of screening (21).

Evaluating the impact of healthcare provider training to improve TB management has mainly been performed in low-income countries and there are only a few rigorous TB training evaluation studies available. E-learning modules use pre- and post- training

For peer review only - http://bmjopen.bmj.com/site/about/guidelines.xhtml

tests to evaluate acquired knowledge. A GP E-learning module has been developed by TB Alert to enhance knowledge of GPs responsible for screening and treatment of LTBI but the effectiveness of the module has not been formally evaluated.

Routine opt-out testing has proven effective for other diseases (HIV / Hepatitis B, C) (22). Factors affecting successful uptake of screening programmes include how the test is offered, by whom, to whom, and in what setting (23). Pregnant women screened for HIV during pregnancy perceived routine opt-out HIV testing as beneficial for both women and their unborn babies (23). Globally, some countries offer routine screening for TB in pregnancy mainly through symptom screen and sputum examination (15).

To test our hypothesis, we will assess the uptake, feasibility and acceptability of screening an at-risk pregnant migrant population for LTBI at routine antenatal booking visits in secondary care, using opt-out IGRA testing. The results from this feasibility study will allow us to develop a definitive large-scale cluster randomised controlled trial (RCT) evaluating the effectiveness of a LTBI screening in antenatal care, the effectiveness of interventions used to maximise migrant screening for LTBI in pregnancy and to increase uptake of LTBI treatment postpartum.

Methods and analysis

Study protocol

This is a prospective observational feasibility study with nested qualitative research involving three hospitals (The Royal London Hospital, Newham University Hospital and Whipps Cross University Hospital). Study participants will enter the cohort when they attend the antenatal clinic for their booking appointment, after they meet inclusion criteria (Table 1). Midwives will offer LTBI screening as an opt-out IGRA blood test alongside other routine investigations for blood borne viruses at the initial booking appointment. The study will assume valid implied consent for participation if women undertake an IGRA test at the time it is offered by the midwife on an opt-out basis. Participants will leave the cohort 6 weeks post-delivery of the baby or at the time of miscarriage if they have had a miscarriage.

At the time of offer of LTBI screening, we will record routine clinical data of all eligible pregnant migrant women including those who do not accept screening. Data on age, ethnicity, year of entry to the UK, pre-existing medical conditions and antenatal history which is routinely recorded in the medical notes will be collected.

All eligible pregnant migrant women will be screened for active TB by their midwives using a standardised symptom assessment questionnaire that includes the WHO recommended TB symptoms screen during their booking appointment. Study participants with a positive IGRA blood test will undergo screening for active TB using the WHO recommended TB symptoms screen at 20 weeks, 30-34 weeks, delivery and post-partum. Data on symptoms of active TB will be collected at each time point (see Figure 1).

All eligible pregnant women will be asked to complete a short questionnaire on acceptability of LTBI screening, knowledge about TB/LTBI, and barriers to screening. At the end of pregnancy, women will be asked to complete the same questionnaire to

For peer review only - http://bmjopen.bmj.com/site/about/guidelines.xhtml

compare the perception and knowledge of active TB/LTBI before and after the screening intervention. Trained research personnel will obtain written informed consent from the participant for the questionnaire.

We have used the SPIRIT reporting guidelines for this paper (24).

Outcomes

Our primary outcomes are (i) the uptake of screening for LTBI in antenatal care assessed by the proportion of eligible migrant women offered a test who accepted LTBI screening, and (ii) the offer of IGRA blood test screening by healthcare providers assessed by the proportion of migrant women eligible for screening who were offered an IGRA test.

Secondary outcomes are: rates of LTBI and active TB identified in pregnant migrant women during the study period, time to diagnosis, understanding and acceptability of LTBI screening and acceptability of interventions to increase screening uptake, perceived facilitators and barriers influencing uptake of LTBI screening and treatment uptake post-partum, increase in knowledge and awareness about active TB/ LTBI amongst pregnant migrant women and healthcare providers and evaluation of cost-effectiveness of LTBI screening in antenatal care compared to primary care.

Process outcomes of the study are the numbers of eligible participants and screening acceptance rate, proportion of eligible pregnant migrant women who were offered LTBI screening, views and experiences of participants on study recruitment methods, data collection methods, and retention in the study and level of NHS support required for the proposed definitive cluster RCT.

Patient and Public Involvement

Healthwatch Newham conducted a survey to evaluate patient experiences of the LTBI screening programme in Newham, to identify the key factors that influence the uptake of screening, and to understand why patients decline screening. The results of this survey have influenced the design of this study, and migrants with LTBI has provided useful information about how LTBI screening could be better conducted. Evaluation of patient experiences demonstrated that migrants would like to be offered a LTBI test directly by their GP or nurse and that the test should be part of a general check-up. Our intervention has been designed to provide this by incorporating the offer of an Interferon-gamma release-assay (IGRA test) into routine antenatal care check–ups by midwives. The concept and the study design has been developed in close collaboration with TB Alert (UK TB charity), with the support of the East London Katherine Twining network PPI group (Katie’s Team) and the Centre for Maternal and Child Health Research at City, University of London’s service user panel and former TB/ LTBI patients. PPI members felt that testing for LTBI as an opt-out approach is an acceptable intervention for pregnant migrant women.

For peer review only - http://bmjopen.bmj.com/site/about/guidelines.xhtml

Sample size

A sample of 200 pregnant migrant women offered testing allows this study to estimate the screening uptake rate (key primary outcome for the feasibility study) with adequate precision across a range of possible values of the rate. If the uptake rate is 50% (at which precision is lowest) then this can be estimated within 6% either side, i.e., a 95% confidence interval of 54-66%. If, however the rate is as high as 80% (or equivalently as low as 20%) then the rate can be estimated within 5%.

Statistical analysis

The primary analysis will focus on estimating the uptake of the screening programme along with the corresponding 95% confidence interval. Secondary analysis will focus on estimating the test positivity. Associations between uptake and potential explanatory variables will be assessed using the Chi squared test, and the strength of association will be presented as an odds ratio with 95% confidence interval. Identification of which characteristics are associated after adjusting for others will be performed using multiple logistic regression, and adjusted odds ratios will be presented.

Nested qualitative research

Study participants will be invited to participate in semi-structured interviews or focus groups to explore acceptability of LTBI screening in antenatal care, understanding of LTBI amongst eligible pregnant women and health care providers, potential use of educational resources in each of these groups and potential barriers/facilitators to LTBI screening and treatment uptake.

A theoretical framework derived from the literature, survey and demographic data will be used to select a purposive sample to explore a range of relevant opinions and experiences. This will include interviewing women who have taken up screening as well as those who have not, or where this is not practicable, those within communities that might be offered screening. Sample size is guided by data saturation: for thematic analysis of semi-structured interviews this is likely to occur between 10 and 40 participants and for focus groups 24-32 participants. Trained research personnel will obtain written informed consent from the participant for the semi-structured interviews and focus groups.

Study participants will be invited to take part in two interviews. The first will take place early in the study (see Figure 1) and will explore participants' understanding of LTBI, along with perceived acceptability of the study and intervention, participants' perceptions of their own risk of TB, their understanding of the prevention of TB and their views on the opt-out screening. The interview will also explore factors that influence participants' decision to be screened and suggestions for what might motivate them or other women to be screened, and their perspectives on the study

For peer review only - http://bmjopen.bmj.com/site/about/guidelines.xhtml

data collection methods. Furthermore, participants' views and attitudes to LTBI treatment during or immediately after pregnancy will be assessed.

A second follow-up interview will take place towards the end of the study (see Figure 1) with those participants who test IGRA positive to discuss their response to receiving a positive screening result, feelings around future treatment and explore what factors might encourage/ discourage women from taking up treatment post-partum. Themes and concepts identified from the first set of interviews will inform the topics raised in the second interviews. This iterative approach will allow follow-up interviews to build on and explore further the participant experience, and to incorporate issues raised by other participants.

Women who decline participation in LTBI screening will be asked by recruiting midwives whether they consent for an independent researcher to contact them for an interview to explore their views. If few 'declining' women consent, up to three community-based focus groups will be conducted with migrant women of childbearing ages, and if appropriate men, in relevant populations to explore their awareness and their views about screening.

Semi-structured interviews will also be conducted with 6-8 healthcare providers, including those who are involved in delivering the intervention, those who have expertise in managing pregnant women and local GPs to whom pregnant women may seek advice about screening and treatment for LTBI.

Two further focus groups with midwives, physicians and nurses, each involving around 8-12 participants will add a different perspective to that of the women. Their views and experiences on approaches to screening for TB/ LTBI in antenatal care, along with perceived barriers/ facilitators to LTBI screening and treatment, from a service or community perspective, will be explored.

Interview and focus group data will be analysed thematically, using constant comparison techniques, to identify, interpret and report patterns (themes) representing beliefs and experiences that participants share (or differ on) in relation to the research questions. The interviews and focus groups will also assess the views and experiences of participants and healthcare providers on study recruitment methods, data collection methods, facilitators and barriers to involvement, and compliance to study procedures.

Data management

All study data will be managed according to the Clinical Effectiveness Group (CEG) data management policy. Data will be entered directly onto a purpose-built database where possible (paper CRFs will be used as a backup if required).

Source data will be taken from the women's antenatal records and entered directly onto a database. Questionnaire data will be generated directly and then entered into the database.

For peer review only - http://bmjopen.bmj.com/site/about/guidelines.xhtml

The Investigator will ensure that patient anonymity is protected and maintained. They will also ensure that patient identities are protected from any unauthorised parties. Information with regards to study patients will be kept confidential and managed in accordance with the Data Protection Act, NHS Caldicott Guardian, The Research Governance Framework for Health and Social Care and Research Ethics Committee Approval.

The study will collect personal data and information about the participants either directly or from their clinical team. Routine clinical data will be entered onto a secure computer database, either by the research team or directly via a secure internet connection. The data will be pseudoanonymised. Any data processed by those outside the research team (research registrar, nurse or project coordinator) will be anonymised. All personal information obtained for the study will be held securely and treated as (strictly) confidential. All staff share the same duty of care to prevent unauthorised disclosure of personal information. No data that could be used to identify an individual will be published.

Transcripts from interviews and focus groups will be archived securely and audio-records destroyed securely following study closure in accordance with City, University of London's data management and retention policy. As all transcripts are de-identified at transcription stage to ensure confidentiality, and personal data will be securely destroyed one year after study closure, no personal data will be included in archived records.

Ethics and Dissemination:

The study has received approval from The Health Research Authority (IRAS 247388) and London- City & East Research Ethics Committee (19/LO/0557). The study has been registered with clinicaltrials.gov (NCT04098341, pre-results). The results will be made available locally to antenatal clinics and primary care physicians, nationally to NHS England and Public Health England and internationally through conferences and journals.

Discussion

Systematic national implementation of the LTBI screening programme is essential to achieving the aims of the collaborative strategy and support the WHO goal of TB elimination. The uptake of LTBI screening amongst migrants is low. This study seeks to provide patient-centred, migrant-inclusive evidence of the uptake, feasibility and acceptability of routine opt-out LTBI screening amongst pregnant migrants in antenatal care. It also seeks to understand potential facilitators and barriers from a healthcare provider perspective. We will assess whether this site of screening results in higher rates of LTBI screening uptake. The results of this study will inform the design of a cluster RCT l trial evaluating the effectiveness of acceptable interventions to maximise migrant screening for LTBI in pregnancy, and to increase uptake of LTBI treatment postpartum.

For peer review only

Funding

This study has been funded by National Institute of Health Research (NIHR) Research for patient benefit programme (Funding reference: PB-PG-0317-20039).

Acknowledgments

We thank all the individuals who participated in this study. We also thank National Institute of Health Research (Funding reference: PB-PG-0317-20039), Health Research Authority (IRAS 247388) and London- City & East Research Ethics Committee (reference: 19/LO/0557).

For peer review only

References

1. Tuberculosis fact sheet. World Health Organisation (WHO). 2019.
2. Houben RM, Dodd PJ. The Global Burden of Latent Tuberculosis Infection: A Re-estimation Using Mathematical Modelling. PLoS Med. 2016;13(10):e1002152.
3. Organisation TWH. End TB Strategy. 2015.
4. Alsdurf H, Hill PC, Matteelli A, Getahun H, Menzies D. The cascade of care in diagnosis and treatment of latent tuberculosis infection: a systematic review and meta-analysis. Lancet Infect Dis. 2016;16(11):1269-78.
5. Kane M, Alter M, Esteban R, Rizzetto M. Role of screening in prevention and treatment. Gut. 1993;34(2 Suppl):S45-7.
6. Llewelyn M, Cropley I, Wilkinson RJ, Davidson RN. Tuberculosis diagnosed during pregnancy: a prospective study from London. Thorax. 2000;55(2):129-32.
7. Asuquo B, Vellore AD, Walters G, Manney S, Mignini L, Kunst H. A case-control study of the risk of adverse perinatal outcomes due to tuberculosis during pregnancy. J Obstet Gynaecol. 2012;32(7):635-8.
8. Zenner D, Kruijshaar ME, Andrews N, Abubakar I. Risk of tuberculosis in pregnancy: a national, primary care-based cohort and self-controlled case series study. Am J Respir Crit Care Med. 2012;185(7):779-84.
9. Zenner D. Crisis-Affected Populations and Tuberculosis. Microbiol Spectr. 2017;5(1).
10. Sobhy S, Babiker Z, Zamora J, Khan KS, Kunst H. Maternal and perinatal mortality and morbidity associated with tuberculosis during pregnancy and the postpartum period: a systematic review and meta-analysis. Bjog. 2017;124(5):727-33.
11. Tuberculosis in England: Annual Report 2019. Public Health England. 2020.
12. Abubakar I, Thomas HL, Morgan M, Anderson S, Zenner D, Cosford P. A collaborative strategy to tackle tuberculosis in England. Lancet. 2015;385(9965):312-3.
13. England PH. Collaborative Tuberculosis Strategy for England 2015-2020.
14. Zenner D, Loutet MG, Harris R, Wilson S, Ormerod LP. Evaluating 17 years of latent tuberculosis infection screening in north-west England: a retrospective cohort study of reactivation. Eur Respir J. 2017;50(1).
15. Montoy JC, Dow WH, Kaplan BC. Patient choice in opt-in, active choice, and opt-out HIV screening: randomized clinical trial. Bmj. 2016;532:h6895.

16. Miele K, Bamrah Morris S, Tepper NK. Tuberculosis in Pregnancy. Obstet Gynecol. 2020;135(6):1444-53.
17. Nguyen HT, Pandolfini C, Chiodini P, Bonati M. Tuberculosis care for pregnant women: a systematic review. BMC Infect Dis. 2014;14:617.
18. Leber W, McMullen H, Anderson J, Marlin N, Santos AC, Bremner S, et al. Promotion of rapid testing for HIV in primary care (RHIVA2): a cluster-randomised controlled trial. Lancet HIV. 2015;2(6):e229-35.
19. Young SD, Monin B, Owens D. Opt-out testing for stigmatized diseases: a social psychological approach to understanding the potential effect of recommendations for routine HIV testing. Health Psychol. 2009;28(6):675-81.
20. Wu S, Roychowdhury I, Khan M. Evaluating the impact of healthcare provider training to improve tuberculosis management: a systematic review of methods and outcome indicators used. Int J Infect Dis. 2017;56:105-10.
21. Loutet MG, Burman M, Jayasekera N, Trathen D, Dart S, Kunst H, Zenner D. National Roll-out of Latent Tuberculosis Testing and Treatment for New Migrants in England: a retrospective evaluation in a high-incidence area. Eur Respir J acceped for publication. 2017.
22. Merchant RC, Waxman MJ. HIV screening in health care settings: some progress, even more questions. Jama. 2010;304(3):348-9.
23. Griffiths C, Sturdy P, Brewin P, Bothamley G, Eldridge S, Martineau A, et al. Educational outreach to promote screening for tuberculosis in primary care: a cluster randomised controlled trial. Lancet. 2007;369(9572):1528-34.
24. Chan AW, Tetzlaff JM, Gøtzsche PC, Altman DG, Mann H, Berlin JA, et al. SPIRIT 2013 explanation and elaboration: guidance for protocols of clinical trials. Bmj. 2013;346:e7586.

Authors' contributions

HK, ST, CMcC, AC, ZD, PJW, CG and IA designed the study and secured funding. AR trained healthcare providers and recruited participants for the study. AR wrote the first draft of the manuscript. All other authors reviewed drafts of this manuscript and commented upon them.

Conflict of Interest

PJW acknowledges funding from the MRC Centre for Global Infectious Disease Analysis (grant number MR/R015600/1); this award is jointly funded by the MRC and Foreign, Commonwealth and Development Office (FCDO) under the MRC/FCDO Concordat agreement and is also part of the European and Developing Countries Clinical Trials Partnership (EDCTP2) programme supported by the EU. PJW is also supported by the NIHR Health Protection Research Unit (HPRU) in Modelling and Health Economics, which is a partnership between Public Health England (PHE), Imperial College London, and LSHTM (grant code NIHR200908). The views expressed are those of the authors and not necessarily those of the UK Department of Health and Social Care, FCDO, EU, MRC, NIHR, or PHE.

Word count: 3661

For peer review only

For peer review only

A feasibility study evaluating the uptake, effectiveness and acceptability of routine screening of pregnant migrants for latent tuberculosis infection in antenatal care: a research protocol

Table 1: Inclusion and exclusion criteria

Inclusion criteria	Exclusion criteria
- Pregnant migrant women aged 16-35 years<br>AND<br>- from high TB incidence countries (incidence of TB of >150/100,000 including sub-Saharan Africa)<br>AND<br>- who have been in the UK for less than 5 years	- Previous history of TB or LTBI<br>- Individuals who are unable to consent<br>- Evidence of current active TB (based of history, examination, blood tests, chest X-ray findings or other radiological findings)

For peer review only

Figure 1. Timeline of study project (Assessment and follow-up of migrant women)

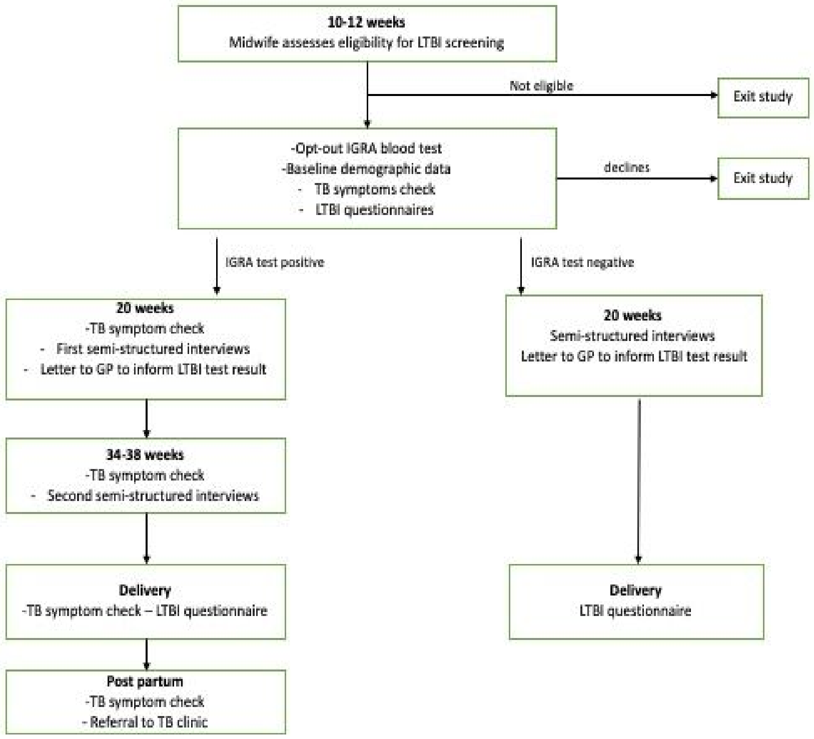
10-12 weeks
Midwife assesses eligibility for LTBI screening
Not eligible
Exit study
-Opt-out IGRA blood test
-Baseline demographic data
- TB symptoms check
- LTBI questionnaires
declines
Exit study
IGRA test positive
IGRA test negative
20 weeks
-TB symptom check
- First semi-structured interviews
- Letter to GP to inform LTBI test result
20 weeks
Semi-structured interviews
Letter to GP to inform LTBI test result
34-38 weeks
-TB symptom check
- Second semi-structured interviews
Delivery
-TB symptom check – LTBI questionnaire
Delivery
LTBI questionnaire
Post partum
-TB symptom check
- Referral to TB clinic

Ymchwil Iechyd a Gofal Cymru
Health and Care Research Wales

NHS
Health Research Authority

Dr Heinke Kunst
Senior Lecturer
Queen Mary University
Blizard Institute
4 Newark Street
London
E1 2AT

Email: hra.approval@nhs.net
Research-permissions@wales.nhs.uk

29 April 2019

Dear Dr Kunst

HRA and Health and Care Research Wales (HCRW) Approval Letter

**Study title:**	**Uptake, effectiveness and acceptability of routine screening of pregnant migrants for latent tuberculosis infection in antenatal care: a feasibility study**
**IRAS project ID:**	**247388**
**REC reference:**	**19/LO/0557**
**Sponsor**	**Queen Mary University of London**

I am pleased to confirm that **HRA and Health and Care Research Wales (HCRW) Approval** has been given for the above referenced study, on the basis described in the application form, protocol, supporting documentation and any clarifications received. You should not expect to receive anything further relating to this application.

Please now work with participating NHS organisations to confirm capacity and capability, in line with the instructions provided in the "Information to support study set up" section towards the end of this letter.

How should I work with participating NHS/HSC organisations in Northern Ireland and Scotland?

HRA and HCRW Approval does not apply to NHS/HSC organisations within Northern Ireland and Scotland.

If you indicated in your IRAS form that you do have participating organisations in either of these devolved administrations, the final document set and the study wide governance report (including this letter) have been sent to the coordinating centre of each participating nation. The relevant national coordinating function/s will contact you as appropriate.

For peer review only - http://bmjopen.bmj.com/site/about/guidelines.xhtml

Please see IRAS Help for information on working with NHS/HSC organisations in Northern Ireland and Scotland.

How should I work with participating non-NHS organisations?
HRA and HCRW Approval does not apply to non-NHS organisations. You should work with your non-NHS organisations to obtain local agreement in accordance with their procedures.

What are my notification responsibilities during the study?

The document "*After Ethical Review – guidance for sponsors and investigators*", issued with your REC favourable opinion, gives detailed guidance on reporting expectations for studies, including:

- Registration of research
- Notifying amendments
- Notifying the end of the study

The HRA website also provides guidance on these topics, and is updated in the light of changes in reporting expectations or procedures.

Who should I contact for further information?
Please do not hesitate to contact me for assistance with this application. My contact details are below.

Your IRAS project ID is **247388**. Please quote this on all correspondence.

Yours sincerely,

Kevin Ahmed
HRA Approvals Manager

Telephone: 0207 104 8171
Email: hra.approval@nhs.net

Copy to: *Dr Mays Jawad, Sponsor Contact, Queen Mary University of London*

For peer review only

List of Documents

The final document set assessed and approved by HRA and HCRW Approval is listed below.

Document	*Version*	*Date*
Evidence of Sponsor insurance or indemnity (non NHS Sponsors only)		27 July 2018
GP/consultant information sheets or letters [GP letter]	1	01 March 2019
HRA Schedule of Events	1.0	05 April 2019
HRA Statement of Activities	1.0	05 April 2019
IRAS Application Form [IRAS_Form_04032019]		04 March 2019
Letter from funder [Intent to Fund]		23 March 2018
Non-validated questionnaire [Knowledge questionnaire]	1	01 March 2019
Other [HP quiz]	1	01 March 2019
Other [Poster]	1	01 March 2019
Participant consent form [Healthcare Workers Interview]	2.0	15 April 2019
Participant consent form [Healthcare Workers Knowledge Questionnaire]	2.0	15 April 2019
Participant consent form [Patient Focus Groups]	2.0	15 April 2019
Participant consent form [Patient Interviews]	2.0	15 April 2019
Participant consent form [Focus Groups]	2.0	15 April 2019
Participant consent form [Patient Questionnaires]	2.0	15 April 2019
Participant information sheet (PIS) [PIS for pregnant women]	1	01 March 2019
Participant information sheet (PIS) [Healthcare Workers]	2.0	15 April 2019
Participant information sheet (PIS) [Patients]	2.0	15 April 2019
Research protocol or project proposal	2.0	15 April 2019
Summary CV for Chief Investigator (CI) [HK CV]	Version 1	03 March 2019

For peer review only

IRAS project ID	247388

Information to support study set up

The below provides all parties with information to support the arranging and confirming of capacity and capability with participating NHS organisations in England and Wales. This is intended to be an accurate reflection of the study at the time of issue of this letter.

Types of participating NHS organisation	Expectations related to confirmation of capacity and capability	Agreement to be used	Funding arrangements	Oversight expectations	HR Good Practice Resource Pack expectations
There is only one participating NHS organisation therefore there is only one site type.	Research activities should not commence at participating NHS organisations in England or Wales prior to their formal confirmation of capacity and capability to deliver the study.	A statement of activities has been submitted and the sponsor is not requesting and does not expect any other site agreement to be used.	No study funding will be provided to sites as per the statement of activities	A Principal Investigator should be appointed at study sites	Where arrangements are not already in place, research staff not employed by the NHS host organisation undertaking any of the research activities listed in the research application would be expected to obtain a Letter of Access based on standard DBS checks and occupational health clearance.

Other information to aid study set-up and delivery

This details any other information that may be helpful to sponsors and participating NHS organisations in England and Wales in study set-up.
Queen Mary Innovation Ltd is the responsible for the commercialisation and management of Barts Health NHS Trust's intellectual property.

For peer review only

NHS
Health Research Authority

London - City & East Research Ethics Committee
Bristol Research Ethics Committee Centre
Whitefriars
Level 3, Block B
Lewins Mead
Bristol
BS1 2NT

<u>Please note</u>: This is the favourable opinion of the REC only and does not allow you to start your study at NHS sites in England until you receive HRA Approval

12 April 2019

Dr Heinke Kunst
Senior Lecturer
Queen Mary University
Blizard Institute
4 Newark Street
London
E1 2AT

Dear Dr Kunst

**Study title:**	**Uptake, effectiveness and acceptability of routine screening of pregnant migrants for latent tuberculosis infection in antenatal care: a feasibility study**
**REC reference:**	**19/LO/0557**
**IRAS project ID:**	**247388**

The Research Ethics Committee reviewed the above application at the meeting held on 04 April 2019. Thank you and Ms Ananna Rahman for attending to discuss the application.

We plan to publish your research summary wording for the above study on the HRA website, together with your contact details. Publication will be no earlier than three months from the date of this favourable opinion letter. The expectation is that this information will be published for all studies that receive an ethical opinion but should you wish to provide a substitute contact point, wish to make a request to defer, or require further information, please contact hra.studyregistration@nhs.net outlining the reasons for your request. Under very limited circumstances (e.g. for student research which has received an unfavourable opinion), it may be possible to grant an exemption to the publication of the study.

Ethical opinion

For peer review only

The members of the Committee present gave a favourable ethical opinion of the above research on the basis described in the application form, protocol and supporting documentation, subject to the conditions specified below. .

Management permission must be obtained from each host organisation prior to the start of the study at the site concerned.

Management permission should be sought from all NHS organisations involved in the study in accordance with NHS research governance arrangements. Each NHS organisation must confirm through the signing of agreements and/or other documents that it has given permission for the research to proceed (except where explicitly specified otherwise).

Guidance on applying for HRA and HCRW Approval (England and Wales)/ NHS permission for research is available in the Integrated Research Application System, at www.hra.nhs.uk or at http://www.rdforum.nhs.uk.

Where a NHS organisation's role in the study is limited to identifying and referring potential participants to research sites ("participant identification centre"), guidance should be sought from the R&D office on the information it requires to give permission for this activity.

For non-NHS sites, site management permission should be obtained in accordance with the procedures of the relevant host organisation.

Sponsors are not required to notify the Committee of management permissions from host organisations.

Registration of Clinical Trials

All clinical trials (defined as the first four categories on the IRAS filter page) must be registered on a publically accessible database. This should be before the first participant is recruited but no later than 6 weeks after recruitment of the first participant.

There is no requirement to separately notify the REC but you should do so at the earliest opportunity e.g. when submitting an amendment. We will audit the registration details as part of the annual progress reporting process.

To ensure transparency in research, we strongly recommend that all research is registered but for non-clinical trials this is not currently mandatory.

If a sponsor wishes to request a deferral for study registration within the required timeframe, they should contact hra.studyregistration@nhs.net. The expectation is that all clinical trials will be registered, however, in exceptional circumstances non registration may be permissible with prior agreement from the HRA. Guidance on where to register is provided on the HRA website.

It is the responsibility of the sponsor to ensure that all the conditions are complied with before the start of the study or its initiation at a particular site (as applicable).

Ethical review of research sites

NHS Sites

For peer review only

The favourable opinion applies to all NHS sites taking part in the study taking part in the study, subject to management permission being obtained from the NHS/HSC R&D office prior to the start of the study (see "Conditions of the favourable opinion" below).

Non NHS sites

The Committee has not yet completed any site-specific assessment(s) (SSA) for the non-NHS research site(s) taking part in this study. The favourable opinion does not therefore apply to any non-NHS site at present. I will write to you again as soon as an SSA application(s) has been reviewed. In the meantime no study procedures should be initiated at non-NHS sites.

Approved documents

The documents reviewed and approved at the meeting were:

Document	*Version*	*Date*
Evidence of Sponsor insurance or indemnity (non NHS Sponsors only)		27 July 2018
GP/consultant information sheets or letters [GP letter]	1	01 March 2019
Initial Assessment for REC		21 March 2019
IRAS Application Form [IRAS_Form_04032019]		04 March 2019
Non-validated questionnaire [Knowledge questionnaire]	1	01 March 2019
Other [PIS for healthcare workers]	1	01 March 2019
Other [Consent form interviews]	1	01 March 2019
Other [Consent form focus groups]	1	01 March 2019
Other [Consent form interviews healthcare workers]	1	01 March 2019
Other [Consent form knowledge questionnaires healthcare workers]	1	01 March 2019
Other [Consent form focus groups healthcare workers]	1	01 March 2019
Other [HP quiz]	1	01 March 2019
Other [Poster]	1	01 March 2019
Participant consent form [Consent form]	1	01 March 2019
Participant information sheet (PIS) [PIS for pregnant women]	1	01 March 2019
Research protocol or project proposal [Protocol]	1	01 March 2019
Summary CV for Chief Investigator (CI) [HK CV]	Version 1	03 March 2019

Membership of the Committee

The members of the Ethics Committee who were present at the meeting are listed on the attached sheet.

The Committee is constituted in accordance with the Governance Arrangements for Research Ethics Committees and complies fully with the Standard Operating Procedures for Research Ethics Committees in the UK.

After ethical review

For peer review only

Reporting requirements

The attached document “After ethical review – guidance for researchers” gives detailed guidance on reporting requirements for studies with a favourable opinion, including:

- Notifying substantial amendments
- Adding new sites and investigators
- Notification of serious breaches of the protocol
- Progress and safety reports
- Notifying the end of the study

The HRA website also provides guidance on these topics, which is updated in the light of changes in reporting requirements or procedures.

User Feedback

The Health Research Authority is continually striving to provide a high quality service to all applicants and sponsors. You are invited to give your view of the service you have received and the application procedure. If you wish to make your views known please use the feedback form available on the HRA website: http://www.hra.nhs.uk/about-the-hra/governance/quality-assurance/

HRA Learning

We are pleased to welcome researchers and research staff to our HRA Learning Events and online learning opportunities– see details at: https://www.hra.nhs.uk/planning-and-improving-research/learning/

**19/LO/0557**	**Please quote this number on all correspondence**

With the Committee’s best wishes for the success of this project.

Yours sincerely

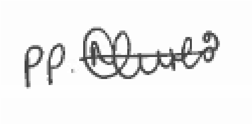

Dr John Keen
Chair

E-mail: nrescommittee.london-cityandeast@nhs.net

Enclosures: *List of names and professions of members who were present at the meeting and those who submitted written comments*

“After ethical review – guidance for researchers”

Copy to: *Dr Mays Jawad, Queen Mary University of London*

Lead Nation
England: HRA.Approval@nhs.net

For peer review only

London - City & East Research Ethics Committee

Attendance at Committee meeting on 04 April 2019

Committee Members:

Name	*Profession*	*Present*	*Notes*
Dr Marie E Bardsley	Director (Pharmaceutical Publication)	Yes	
Ms Clare Barron	Solicitor – in-house legal department	No	
Dr Ayse Baxter	Pharmaceutical Physician	No	
Dr Luis Beltran	Consultant Histopathologist	No	
Ms Ann Black	Health Economist/Psycho-social Researcher	Yes	
Mr Frank Cross	Consultant General and Vascular Surgeon	Yes	
Mr Fasahat Hussain		Yes	
Mrs Lisa Johnson	Head of Clinical Operations	Yes	
Dr John Keen	GP (REC Chairman)	Yes	
Mr Rajat Khullar	REC Manager	No	
Dr Kieran McCafferty	Nephrologist Consultant/ Hon Senior Lecturer	No	
Dr Paul Metcalfe	Research Scientist - immunohaematology	Yes	
Ms Anna (Renqian) Song	Pharmacist	No	

Also in attendance:

Name	*Position (or reason for attending)*
Mr Kevin Ahmed	Approvals Manager
Miss Nicole Curtis	Approvals Specialist
Mrs Isabel Moldon	Observer
Mrs Frances Sarah Sharratt	Observer

For peer review only

Reporting checklist for protocol of a clinical trial.

Based on the SPIRIT guidelines.

Instructions to authors

Complete this checklist by entering the page numbers from your manuscript where readers will find each of the items listed below.

Your article may not currently address all the items on the checklist. Please modify your text to include the missing information. If you are certain that an item does not apply, please write "n/a" and provide a short explanation.

Upload your completed checklist as an extra file when you submit to a journal.

In your methods section, say that you used the SPIRITreporting guidelines, and cite them as:

Chan A-W, Tetzlaff JM, Gøtzsche PC, Altman DG, Mann H, Berlin J, Dickersin K, Hróbjartsson A, Schulz KF, Parulekar WR, Krleža-Jerić K, Laupacis A, Moher D. SPIRIT 2013 Explanation and Elaboration: Guidance for protocols of clinical trials. BMJ. 2013;346:e7586

		Reporting Item	Page Number
**Administrative information**			
Title	1	Descriptive title identifying the study design, population, interventions, and, if applicable, trial acronym	
Trial registration	2	Trial identifier and registry name. If not yet registered, name of intended registry	
Trial registration: data set	NA	All items from the World Health Organization Trial Registration Data Set	
Protocol version	NA	Date and version identifier	
Funding	9	Sources and types of financial, material, and other support	
Roles and responsibilities: contributorship	11	Names, affiliations, and roles of protocol contributors	

For peer review only

Roles and responsibilities: sponsor contact information	NA	Name and contact information for the trial sponsor
Roles and responsibilities: sponsor and funder	NA	Role of study sponsor and funders, if any, in study design; collection, management, analysis, and interpretation of data; writing of the report; and the decision to submit the report for publication, including whether they will have ultimate authority over any of these activities
Roles and responsibilities: committees	NA	Composition, roles, and responsibilities of the coordinating centre, steering committee, endpoint adjudication committee, data management team, and other individuals or groups overseeing the trial, if applicable (see Item 21a for data monitoring committee)
**Introduction**		
Background and rationale	3-5	Description of research question and justification for undertaking the trial, including summary of relevant studies (published and unpublished) examining benefits and harms for each intervention
Background and rationale: choice of comparators	NA	Explanation for choice of comparators
Objectives	4-5	Specific objectives or hypotheses
Trial design	5-6	Description of trial design including type of trial (eg, parallel group, crossover, factorial, single group), allocation ratio, and framework (eg, superiority, equivalence, non-inferiority, exploratory)
**Methods: Participants, interventions, and outcomes**		
Study setting	5-6	Description of study settings (eg, community clinic, academic hospital) and list of countries where data will be collected. Reference to where list of study sites can be obtained

For peer review only

Eligibility criteria	5-6	Inclusion and exclusion criteria for participants. If applicable, eligibility criteria for study centres and individuals who will perform the interventions (eg, surgeons, psychotherapists)
Interventions: description	5-6	Interventions for each group with sufficient detail to allow replication, including how and when they will be administered
Interventions: modifications	NA	Criteria for discontinuing or modifying allocated interventions for a given trial participant (eg, drug dose change in response to harms, participant request, or improving / worsening disease)
Interventions: adherance	NA	Strategies to improve adherence to intervention protocols, and any procedures for monitoring adherence (eg, drug tablet return; laboratory tests)
Interventions: concomitant care	NA	Relevant concomitant care and interventions that are permitted or prohibited during the trial
Outcomes	6	Primary, secondary, and other outcomes, including the specific measurement variable (eg, systolic blood pressure), analysis metric (eg, change from baseline, final value, time to event), method of aggregation (eg, median, proportion), and time point for each outcome. Explanation of the clinical relevance of chosen efficacy and harm outcomes is strongly recommended
Participant timeline	Figure 1	Time schedule of enrolment, interventions (including any run-ins and washouts), assessments, and visits for participants. A schematic diagram is highly recommended (see Figure)
Sample size	7	Estimated number of participants needed to achieve study objectives and how it was determined, including clinical and statistical assumptions supporting any sample size calculations
Recruitment	5-6	Strategies for achieving adequate participant enrolment to reach target sample size
**Methods: Assignment of interventions (for controlled trials)**		
Allocation: sequence generation	NA	Method of generating the allocation sequence (eg, computer-generated random numbers), and list of any factors for stratification. To reduce predictability of a random sequence, details of any planned restriction (eg, blocking) should be

For peer review only

		provided in a separate document that is unavailable to those who enrol participants or assign interventions
Allocation concealment mechanism	NA	Mechanism of implementing the allocation sequence (eg, central telephone; sequentially numbered, opaque, sealed envelopes), describing any steps to conceal the sequence until interventions are assigned
Allocation: implementation	NA	Who will generate the allocation sequence, who will enrol participants, and who will assign participants to interventions
Blinding (masking)	NA	Who will be blinded after assignment to interventions (eg, trial participants, care providers, outcome assessors, data analysts), and how
Blinding (masking): emergency unblinding	NA	If blinded, circumstances under which unblinding is permissible, and procedure for revealing a participant's allocated intervention during the trial
**Methods: Data collection, management, and analysis**		
Data collection plan	8-9	Plans for assessment and collection of outcome, baseline, and other trial data, including any related processes to promote data quality (eg, duplicate measurements, training of assessors) and a description of study instruments (eg, questionnaires, laboratory tests) along with their reliability and validity, if known. Reference to where data collection forms can be found, if not in the protocol
Data collection plan: retention	8-9	Plans to promote participant retention and complete follow-up, including list of any outcome data to be collected for participants who discontinue or deviate from intervention protocols
Data management	8-9	Plans for data entry, coding, security, and storage, including any related processes to promote data quality (eg, double data entry; range checks for data values). Reference to where details of data management procedures can be found, if not in the protocol

For peer review only

Statistics: outcomes	7	Statistical methods for analysing primary and secondary outcomes. Reference to where other details of the statistical analysis plan can be found, if not in the protocol
Statistics: additional analyses	7	Methods for any additional analyses (eg, subgroup and adjusted analyses)
Statistics: analysis population and missing data	NA	Definition of analysis population relating to protocol non-adherence (eg, as randomised analysis), and any statistical methods to handle missing data (eg, multiple imputation)
**Methods: Monitoring**		
Data monitoring: formal committee	NA	Composition of data monitoring committee (DMC); summary of its role and reporting structure; statement of whether it is independent from the sponsor and competing interests; and reference to where further details about its charter can be found, if not in the protocol. Alternatively, an explanation of why a DMC is not needed
Data monitoring: interim analysis	NA	Description of any interim analyses and stopping guidelines, including who will have access to these interim results and make the final decision to terminate the trial
Harms	NA	Plans for collecting, assessing, reporting, and managing solicited and spontaneously reported adverse events and other unintended effects of trial interventions or trial conduct
Auditing	NA	Frequency and procedures for auditing trial conduct, if any, and whether the process will be independent from investigators and the sponsor
**Ethics and dissemination**		
Research ethics approval	9	Plans for seeking research ethics committee / institutional review board (REC / IRB) approval
Protocol amendments	NA	Plans for communicating important protocol modifications (eg, changes to eligibility criteria, outcomes, analyses) to relevant parties (eg, investigators, REC / IRBs, trial participants, trial registries, journals, regulators)

For peer review only

Consent or assent	NA	Who will obtain informed consent or assent from potential trial participants or authorised surrogates, and how (see Item 32)
Consent or assent: ancillary studies	NA	Additional consent provisions for collection and use of participant data and biological specimens in ancillary studies, if applicable
Confidentiality	8-9	How personal information about potential and enrolled participants will be collected, shared, and maintained in order to protect confidentiality before, during, and after the trial
Declaration of interests	11	Financial and other competing interests for principal investigators for the overall trial and each study site
Data access	8-9	Statement of who will have access to the final trial dataset, and disclosure of contractual agreements that limit such access for investigators
Ancillary and post trial care	NA	Provisions, if any, for ancillary and post-trial care, and for compensation to those who suffer harm from trial participation
Dissemination policy: trial results	9	Plans for investigators and sponsor to communicate trial results to participants, healthcare professionals, the public, and other relevant groups (eg, via publication, reporting in results databases, or other data sharing arrangements), including any publication restrictions
Dissemination policy: authorship	NA	Authorship eligibility guidelines and any intended use of professional writers
Dissemination policy: reproducible research	NA	Plans, if any, for granting public access to the full protocol, participant-level dataset, and statistical code
**Appendices**		
Informed consent materials	NA	Model consent form and other related documentation given to participants and authorised surrogates
Biological specimens	NA	Plans for collection, laboratory evaluation, and storage of biological specimens for genetic or molecular analysis in the current trial and for future use in ancillary studies, if applicable

None The SPIRIT Explanation and Elaboration paper is distributed under the terms of the Creative Commons Attribution License CC-BY-NC. This checklist can be completed online using https://www.goodreports.org/, a tool made by the EQUATOR Network in collaboration with Penelope.ai

For peer review only

BMJ Open

A feasibility study evaluating the uptake, effectiveness and acceptability of routine screening of pregnant migrants for latent tuberculosis infection in antenatal care: a research protocol

Journal:	*BMJ Open*
Manuscript ID	bmjopen-2021-058734.R1
Article Type:	Protocol
Date Submitted by the Author:	15-Feb-2022

Complete List of Authors:	Rahman, A; Queen Mary University of London<br>Thangaratinam, Shakila; Queen Mary University of London, BARC (Barts Research Centre for Women's Health), Blizard Institute, Barts and the London School of Medicine and Dentistry<br>Copas, Andrew; UCL<br>Zenner, D; Queen Mary University of London, Institute of Population Health Sciences<br>White, Peter; Imperial College, MRC Centre for Global Infectious Disease Analysis and NIHR Health Protection Research Unit in Modelling and Health Economics; Public Health England, Modelling and Economics Unit<br>Griffiths, Chris; Barts and The London School of Medicine and Dentistry,<br>Abubakar, Ibrahim; Health Protection Agency, Tuberculosis Section, Centre for Infections<br>McCourt, Christine; Department of Midwifery and Child health, City University London<br>Kunst, Heinke; Queen Mary University of London, Department of Respiratory Medicine

**Primary Subject Heading**:	Public health
Secondary Subject Heading:	Infectious diseases, Reproductive medicine, Respiratory medicine
Keywords:	Antenatal < GENETICS, Tuberculosis < INFECTIOUS DISEASES, Public health < INFECTIOUS DISEASES

SCHOLARONE™ Manuscripts

A feasibility study evaluating the uptake, effectiveness and acceptability of routine screening of pregnant migrants for latent tuberculosis infection in antenatal care: a research protocol

Rahman A[1], Thangaratinam S[2], Copas A[3], Zenner D[4], White PJ[5,6], Griffiths C[4], Abubaker I[3], McCourt C[7], Kunst H[1]

[1]Blizard Institute, Barts and The London School of Medicine and Dentistry, Queen Mary University of London, London E1 2AT

[2] WHO Collaborating Centre for Women's Health, Institute of Translational Medicine, Heritage Building, Birmingham, B15 2TH

[3] Institute for Global Health, University College London, Mortimer Market Centre, London, WC1E 6JB

[4] Institute of Population Health Sciences, Queen Mary University of London, London E1 2AB

[5] MRC Centre for Global Infectious Disease Analysis and NIHR Health Protection Research Unit in Modelling and Health Economics, School of Public Health, Imperial College London, Norfolk Place, London, W2 1PG, UK

[6] Modelling and Economics Unit, National Infection Service, Public Health England, London, NW9 5EQ, UK

[7] Centre for Maternal & Child Health Research, City, University of London

Corresponding Author

Dr Ananna Rahman,

Blizard Institute,

Barts and The London School of Medicine and Dentistry, Queen Mary University,

4 Newark Street, London E1 2AT, UK.

E-mail: ananna.rahman1@nhs.net

For peer review only

Abstract

Introduction

Globally, Tuberculosis (TB) is a leading cause of death in women of reproductive age and there is high risk of reactivation of latent tuberculosis infection (LTBI) in pregnancy. The uptake of routine screening of migrants for LTBI in the UK in primary care is low. Antenatal care is a novel setting which could improve uptake and can lend insight into the feasibility and acceptability of offering opt-out screening for LTBI.

Methods and analysis

This is an observational feasibility study with a nested qualitative component. The setting will be the antenatal clinics in three hospitals in East London, UK . Inclusion criteria are pregnant migrant women aged 16-35 years attending antenatal clinics who are from countries with a TB incidence of greater than 150/100,000 including sub-Saharan Africa, and who have been in the UK for less than 5 years. Participants will be offered LTBI screening with an opt-out interferon gamma release assay (IGRA) blood test, and be invited to complete a questionnaire. Both participants and healthcare providers will be invited to participate in semi-structured interviews or focus groups to evaluate understanding, feasibility and acceptability of routine opt-out LTBI screening. The primary analysis will focus on estimating the uptake of the screening programme along with the corresponding 95% confidence interval. Secondary analysis will focus on estimating the test positivity. Qualitative analysis will evaluate the acceptability of offering routine opt-out LTBI screening to participants and healthcare providers.

For peer review only - http://bmjopen.bmj.com/site/about/guidelines.xhtml

Ethics and dissemination

The study has received the following approvals: Health Research Authority (IRAS 247388) and National Health Service Ethics Committee (19/LO/0557). The results will be made available locally to antenatal clinics and primary care physicians, nationally to NHS England and Public Health England and internationally through conferences and journals.

For peer review only

Trial Registration Number: NCT04098341, pre-results.

Article Summary

Strengths and limitations of this study:

- The study uses a novel approach of tackling a complex problem of low uptake of latent tuberculosis infection (LTBI) screening in migrants by using an opt-out method and a novel setting: antenatal care
- The study creates new education and training tools for healthcare professionals working in antenatal care
- Our findings will provide a greater understanding of the acceptability of LTBI screening amongst pregnant migrant women and healthcare professionals.
- As this is an observational study, we are unable to demonstrate causality from our results.
- Not being able to interview women who decline LTBI screening may reduce the validity of our findings.

Introduction

Context

Tuberculosis (TB) remains a significant global health problem affecting an estimated 10 million people worldwide in 2019 leading to 1.4 million deaths (1). TB is one of the leading causes of death in women of reproductive age (15-45 years) (2). In 2018, an estimated 3.2 million women globally were infected with TB and almost half a million women died from TB (3). Indirect maternal deaths account for 28% of total maternal deaths, of which 15-35% are due to TB (2).

The World Health Organisation defines LTBI as a 'state of persistent immune response stimulation by *Mycobacterium Tuberculosis* antigens without evidence of clinically manifested active TB' (1). A quarter of the world's population is estimated to have latent TB infection (LTBI) (4). Individuals with LTBI have no signs and symptoms of active TB but remain at risk of developing active TB in their lifetime. LTBI acts as a reservoir for active TB and TB elimination requires strategies for LTBI control (5). The risk of reactivation of LTBI is higher in pregnancy (6). This risk may be due to T-cell suppression and reduced interferon-gamma production (7).

In low TB incidence countries, TB transmission is limited and most active cases of TB occur due to reactivation of LTBI imported from high incidence settings (8). Uptake of LTBI screening in primary care is low (9, 10). Antenatal care is a new setting for LTBI screening and understanding the factors affecting the feasibility and acceptability of LTBI screening in this setting are first steps towards developing effective interventions to improving LTBI screening uptake.

For peer review only - http://bmjopen.bmj.com/site/about/guidelines.xhtml

Current knowledge

In women of childbearing age (16-45years), TB is one of three leading causes of death globally (4). Diagnosis of TB in pregnancy is often delayed as pregnancy can mask some of the clinical manifestations of TB (11, 12). TB in pregnancy is associated with poor perinatal, foetal and maternal outcomes (13, 14).

The UK has one of the highest TB incidence rates in Western Europe. The incidence of TB among those born outside the UK is 14 times higher at 39.0 per 100,000 population and accounting for 74% of all new cases of TB in England in 2019 (15). Public Health England's TB migrant health guide strategy recommends migrant screening for LTBI in high incidence areas in England such as the London Boroughs of Tower Hamlets, Newham and Waltham Forrest (16, 17).

The London Borough of Newham was spearheading a large-scale LTBI screening programme in anticipation of the national programme. A total of 20,905 LTBI tests were reported between July 2014 and June 2017 across England with nearly half of the tests taking place in Newham (9, 10). Between April 2015 and June 2016, 5,622 eligible migrants in England were offered an LTBI test, 2,904 (51%) of whom attended for the test (9).

Effective screening for LTBI is key to reducing TB incidence in the UK. There is good evidence that screening and treatment of LTBI is a cost-effective intervention that significantly reduces the risk of developing active disease and the risk of onward transmission (18, 19). The national LTBI migrant screening programme has been

For peer review only

rolled out but there is insufficient evidence on the best setting for uptake of LTBI screening.

There is limited qualitative research about the acceptability to women of LTBI screening in pregnancy. Reasons for low uptake may be due to stigma of having active TB or fear of a positive test result affecting their immigration status. An opt-out approach to LTBI screening may normalise the process and has the potential to reduce barriers such as stigma, as well as practical barriers (20).

Provider knowledge and understanding of the risks of TB, screening and treatment can be a major predictor of successful management of TB (21). Data from a local LTBI screening programme has highlighted that offer of screening varies amongst GP practices indicating that health care provider knowledge and attitude may influence offer of screening (22).

Evaluating the impact of healthcare provider training to improve TB management has mainly been performed in low-income countries and there are only a few rigorous TB training evaluation studies available (21). E-learning modules use pre- and post-training tests to evaluate acquired knowledge. A GP E-learning module has been developed by the national TB charity “TB Alert” to enhance knowledge of GPs responsible for screening and treatment of LTBI but the effectiveness of the module has not been formally evaluated.

Rationale for LTBI screening in antenatal care

For peer review only - http://bmjopen.bmj.com/site/about/guidelines.xhtml

Pregnancy can predispose to reactivation of LTBI and diagnosis can be delayed due to reduced awareness among healthcare providers and reluctance to investigate non-specific TB symptoms by chest radiography (23). Risks of LTBI reactivation and delays in diagnosis of TB can be mitigated by screening an at-risk pregnant migrant population for LTBI. A simple clinical algorithm recommended by the WHO based on absence of current cough, fever, weight loss, and night sweats can help to exclude active TB disease. Moreover, healthcare professionals will have a higher index of suspicion for active TB in IGRA positive pregnant migrant women presenting with symptoms suggestive of TB, thus preventing a delay in diagnosis (24).

Pregnant migrants may not be accessing routine health care and often do not have a GP. Antenatal care may therefore be a key opportunity to assess the woman's health and screen for TB. Antenatal care provides an opportunity for health promotion such as advocating GP registration and is a time when parents may be particularly receptive to public health information and promotion.

Routine opt-out testing has proven effective for other diseases (HIV / Hepatitis B, C) (25). Factors affecting successful uptake of screening programmes include how the test is offered, by whom, to whom, and in what setting (26). Pregnant women screened for HIV during pregnancy perceived routine opt-out HIV testing as beneficial for both women and their unborn babies (26). Globally, some countries offer routine screening for TB in pregnancy mainly through symptom screen and sputum examination (19).

For peer review only

LTBI screening for migrants from high TB incidence countries in antenatal care has shown high uptake in the U.S. but feasibility of LTBI screening in antenatal clinics in the UK has not been evaluated (27).

Research hypothesis and aims

We hypothesise that offering routine opt-out LTBI screening to an at-risk pregnant migrant population in antenatal care will be feasible and acceptable to pregnant migrant women and healthcare providers.

To test our hypothesis, we will assess the uptake, feasibility and acceptability of screening an at-risk pregnant migrant population for LTBI at routine antenatal booking visits in secondary care, using opt-out IGRA testing. The results from this feasibility study will allow us to develop a definitive large-scale cluster randomised controlled trial (RCT) evaluating the effectiveness of a LTBI screening in antenatal care, the effectiveness of interventions used to maximise migrant screening for LTBI in pregnancy and to increase uptake of LTBI treatment postpartum.

Methods and analysis

Study protocol

This is a prospective observational feasibility study with nested qualitative research which will take place in antenatal booking clinics of three hospitals in East London (The Royal London Hospital, Newham University Hospital and Whipps Cross University Hospital). The study started on 29th April 2019 and the first participant was recruited on 3rd July 2019. The study is due to finish on 31st May 2022.

For peer review only - http://bmjopen.bmj.com/site/about/guidelines.xhtml

Educational and training tools will be developed before the study begins. Healthcare providers involved in antenatal care will be asked to complete an E-learning module on active TB/LTBI, which has been developed by the study team, along with the national TB charity (TB Alert) and the Royal College of Midwives.

Study participants will enter the cohort when they attend the antenatal clinic for their booking appointment, after they meet inclusion criteria (Table 1). Midwives will counsel and offer LTBI screening as an opt-out IGRA (Interferon gamma release assay) blood test alongside other routine investigations for blood borne viruses at the initial booking appointment. The study will assume valid implied consent for participation if women undertake an IGRA test at the time it is offered by the midwife on an opt-out basis. Participants will be given a Participant Information Sheet by the midwife at this appointment detailing the study. Routine blood tests, including IGRA, will be taken by phlebotomists based in antenatal care.

Table 1: Inclusion and exclusion criteria

Inclusion criteria	Exclusion criteria
- Pregnant migrant women aged 16-35 years<br>AND<br>- from high TB incidence countries (incidence of TB of >150/100,000 including sub-Saharan Africa)<br>AND<br>- who have been in the UK for less than 5 years	- Previous history of TB or LTBI<br>- Individuals who are unable to consent<br>- Evidence of current active TB (based of history, examination, blood tests, chest X-ray findings or other radiological findings)

At the time of offer of LTBI screening, we will record routine clinical data of all eligible pregnant migrant women including those who do not accept screening. Data on age,

For peer review only - http://bmjopen.bmj.com/site/about/guidelines.xhtml

ethnicity, year of entry to the UK, pre-existing medical conditions and antenatal history which is routinely recorded in the medical notes will be collected.

All eligible pregnant migrant women will be screened for active TB by their midwives using a standardised symptom assessment questionnaire that includes the WHO recommended TB symptoms screen during their initial booking appointment. Study participants with a positive IGRA blood test will then undergo screening for active TB using the WHO recommended TB symptoms screen at 20 weeks, 30-34 weeks, delivery and post-partum. Data on symptoms of active TB will be collected at each time point (see Figure 1).

Participants will leave the study 6 weeks post-delivery or at the time of miscarriage if they have had a miscarriage.

Study participants with a positive IGRA blood test will be referred to the local TB clinic (if screened at The Royal London Hospital or Whipps Cross University Hospital) or to their GP (if screened in Newham University Hospital). TB clinics or GPs will review these individuals and initiate LTBI treatment according to local protocols.

All eligible pregnant women will be asked to complete a short questionnaire on acceptability of LTBI screening, knowledge about TB/LTBI, and barriers to screening. At the end of pregnancy, women will be asked to complete the same questionnaire to compare the perception and knowledge of active TB/LTBI before and after the screening intervention. Trained research personnel will obtain written informed consent from the participant for the questionnaire.

For peer review only

We have used the SPIRIT reporting guidelines for this paper (28).

Outcomes

Our primary outcomes are (i) the uptake of screening for LTBI in antenatal care assessed by the proportion of eligible migrant women offered a test who accepted LTBI screening, and (ii) the offer of IGRA blood test screening by healthcare providers assessed by the proportion of migrant women eligible for screening who were offered an IGRA test.

Secondary outcomes are: rates of LTBI and active TB identified in pregnant migrant women during the study period, time to diagnosis, understanding and acceptability of LTBI screening and acceptability of interventions to increase screening uptake, perceived facilitators and barriers influencing uptake of LTBI screening and treatment uptake post-partum, increase in knowledge and awareness about active TB/ LTBI amongst pregnant migrant women and healthcare providers and estimation of some of the parameters required for evaluation of cost-effectiveness of LTBI screening in antenatal care compared to primary care.

Process outcomes of the study are the numbers of eligible participants and screening acceptance rate, proportion of eligible pregnant migrant women who were offered LTBI screening, views and experiences of participants on study recruitment methods, data collection methods, and retention in the study and level of NHS support required for the proposed definitive cluster RCT.

For peer review only

Patient and Public Involvement

Healthwatch Newham conducted a survey to evaluate patient experiences of the LTBI screening programme in Newham, to identify the key factors that influence the uptake of screening, and to understand why patients decline screening. The results of this survey have influenced the design of this study, and migrants with LTBI has provided useful information about how LTBI screening could be better conducted. Evaluation of patient experiences demonstrated that migrants would like to be offered a LTBI test directly by their GP or nurse and that the test should be part of a general check-up. Our intervention has been designed to provide this by incorporating the offer of an Interferon-gamma release-assay (IGRA test) into routine antenatal care check–ups by midwives. The concept and the study design has been developed in close collaboration with TB Alert (UK TB charity), with the support of the East London Katherine Twining network PPI group (Katie's Team) and the Centre for Maternal and Child Health Research at City, University of London's service user panel and former TB/ LTBI patients. PPI members felt that testing for LTBI as an opt-out approach is an acceptable intervention for pregnant migrant women.

Sample size

A sample of 200 pregnant migrant women offered testing allows this study to estimate the screening uptake rate (key primary outcome for the feasibility study) with adequate precision across a range of possible values of the rate. If the uptake rate is 50% (at which precision is lowest) then this can be estimated within 6% either side, i.e., a 95% confidence interval of 44-56%. If, however the rate is as high as 80% (or equivalently as low as 20%) then the rate can be estimated within 5%.

For peer review only - http://bmjopen.bmj.com/site/about/guidelines.xhtml

These precision calculations are based on the standard Normal approximation and formula for a 95% confidence interval for a proportion p based on a sample size n: p ± 1.96 x sqrt[p x (1-p) / n].

Statistical analysis

The primary analysis will focus on estimating the uptake of the screening programme along with the corresponding 95% confidence interval. Secondary analysis will focus on estimating the test positivity. Associations between uptake and potential explanatory variables will be assessed using the Chi squared test, and the strength of association will be presented as an odds ratio with 95% confidence interval. Identification of which characteristics are associated after adjusting for others will be performed using multiple logistic regression, and adjusted odds ratios will be presented.

For peer review only

Nested qualitative research

Study participants will be invited to participate in semi-structured interviews or focus groups to explore acceptability of LTBI screening in antenatal care, understanding of LTBI amongst eligible pregnant women and health care providers, potential use of educational resources in each of these groups and potential barriers/facilitators to LTBI screening and treatment uptake.

A theoretical framework derived from the literature, survey and demographic data will be used to select a purposive sample to explore a range of relevant opinions and experiences. This will include interviewing women who have taken up screening as well as those who have not, or where this is not practicable, those within communities

For peer review only - http://bmjopen.bmj.com/site/about/guidelines.xhtml

that might be offered screening. Sample size is guided by data saturation: for thematic analysis of semi-structured interviews this is likely to occur between 10 and 40 participants and for focus groups 24-32 participants. Trained research personnel will obtain written informed consent from the participant for the semi-structured interviews and focus groups.

Study participants will be invited to take part in two interviews. The first will take place early in the study (see Figure 1) and will explore participants' understanding of LTBI, along with perceived acceptability of the study and intervention, participants' perceptions of their own risk of TB, their understanding of the prevention of TB and their views on the opt-out screening. The interview will also explore factors that influence participants' decision to be screened and suggestions for what might motivate them or other women to be screened, and their perspectives on the study data collection methods. Furthermore, participants' views and attitudes to LTBI treatment during or immediately after pregnancy will be assessed.

A second follow-up interview will take place towards the end of the study (see Figure 1) with those participants who test IGRA positive to discuss their response to receiving a positive screening result, feelings around future treatment and explore what factors might encourage/ discourage women from taking up treatment post-partum. Themes and concepts identified from the first set of interviews will inform the topics raised in the second interviews. This iterative approach will allow follow-up interviews to build on and explore further the participant experience, and to incorporate issues raised by other participants.

For peer review only - http://bmjopen.bmj.com/site/about/guidelines.xhtml

Women who decline participation in LTBI screening will be asked by recruiting midwives whether they consent for an independent researcher to contact them for an interview to explore their views. If few ‘declining’ women consent, up to three community-based focus groups will be conducted with migrant women of childbearing ages, and if appropriate men, in relevant populations to explore their awareness and their views about screening.

Semi-structured interviews will also be conducted with 6-8 healthcare providers, including those who are involved in delivering the intervention, those who have expertise in managing pregnant women and local GPs to whom pregnant women may seek advice about screening and treatment for LTBI.

Two further focus groups with midwives, physicians and nurses, each involving around 8-12 participants will add a different perspective to that of the women. Their views and experiences on approaches to screening for TB/ LTBI in antenatal care, along with perceived barriers/ facilitators to LTBI screening and treatment, from a service or community perspective, will be explored.

Interview and focus group data will be analysed thematically, using constant comparison techniques, to identify, interpret and report patterns (themes) representing beliefs and experiences that participants share (or differ on) in relation to the research questions. The interviews and focus groups will also assess the views and experiences of participants and healthcare providers on study recruitment methods, data collection methods, facilitators and barriers to involvement, and compliance to study procedures.

For peer review only

Data management

All study data will be managed according to the Clinical Effectiveness Group (CEG) data management policy. Data will be entered directly onto a purpose-built database where possible (paper CRFs will be used as a backup if required).

Source data will be taken from the women's antenatal records and entered directly onto a database. Questionnaire data will be generated directly and then entered into the database.

The Investigator will ensure that patient anonymity is protected and maintained. They will also ensure that patient identities are protected from any unauthorised parties. Information with regards to study patients will be kept confidential and managed in accordance with the Data Protection Act, NHS Caldicott Guardian, The Research Governance Framework for Health and Social Care and Research Ethics Committee Approval.

The study will collect personal data and information about the participants either directly or from their clinical team. Routine clinical data will be entered onto a secure computer database, either by the research team or directly via a secure internet connection. The data will be pseudoanonymised. Any data processed by those outside the research team (research registrar, nurse or project coordinator) will be anonymised. All personal information obtained for the study will be held securely and treated as (strictly) confidential. All staff share the same duty of care to prevent unauthorised disclosure of personal information. No data that could be used to identify an individual will be published.

For peer review only - http://bmjopen.bmj.com/site/about/guidelines.xhtml

Transcripts from interviews and focus groups will be archived securely and audio-records destroyed securely following study closure in accordance with City, University of London's data management and retention policy. As all transcripts are de-identified at transcription stage to ensure confidentiality, and personal data will be securely destroyed one year after study closure, no personal data will be included in archived records.

Ethics and Dissemination:

The study has received approval from The Health Research Authority (IRAS 247388) and London- City & East Research Ethics Committee (19/LO/0557). The study has been registered with clinicaltrials.gov (NCT04098341, pre-results). The results will be made available locally to antenatal clinics and primary care physicians, nationally to NHS England and Public Health England and internationally through conferences and journals.

Discussion

Systematic national implementation of the LTBI screening programme is essential to achieving the aims of the collaborative strategy and support the WHO goal of TB elimination. The uptake of LTBI screening amongst migrants is low. This study seeks to provide patient-centred, migrant-inclusive evidence of the uptake, feasibility and acceptability of routine opt-out LTBI screening amongst pregnant migrants in antenatal care. It also seeks to understand potential facilitators and barriers from a healthcare provider perspective. We will assess whether this site of screening results in higher rates of LTBI screening uptake. The results of this study will inform the design of a

For peer review only

cluster RCT trial evaluating the effectiveness of acceptable interventions to maximise migrant screening for LTBI in pregnancy, and to increase uptake of LTBI treatment postpartum.

Funding

This study has been funded by National Institute of Health Research (NIHR) Research for patient benefit programme (Funding reference: PB-PG-0317-20039).

Acknowledgments

We thank all the individuals who participated in this study. We also thank National Institute of Health Research (Funding reference: PB-PG-0317-20039), Health Research Authority (IRAS 247388) and London- City & East Research Ethics Committee (reference: 19/LO/0557).

Authors' contributions

HK, ST, CMcC, AC, ZD, PJW, CG and IA designed the study and secured funding. AR trained healthcare providers and recruited participants for the study. AR wrote the first draft of the manuscript. All other authors reviewed drafts of this manuscript and commented upon them.

Conflict of Interest

PJW acknowledges funding from the MRC Centre for Global Infectious Disease Analysis (grant number MR/R015600/1); this award is jointly funded by the MRC and Foreign, Commonwealth and Development Office (FCDO) under the MRC/FCDO Concordat agreement and is also part of the European and Developing Countries

For peer review only

Clinical Trials Partnership (EDCTP2) programme supported by the EU. PJW is also supported by the NIHR Health Protection Research Unit (HPRU) in Modelling and Health Economics, which is a partnership between Public Health England (PHE), Imperial College London, and LSHTM (grant code NIHR200908). The views expressed are those of the authors and not necessarily those of the UK Department of Health and Social Care, FCDO, EU, MRC, NIHR, or PHE.

For peer review only

References

1. Tuberculosis fact sheet. World Health Organisation (WHO). 2019.
2. Say L, Chou D, Gemmill A, Tunçalp Ö, Moller AB, Daniels J, et al. Global causes of maternal death: a WHO systematic analysis. Lancet Glob Health. 2014;2(6):e323-33.
3. Organisation WH. Tuberculosis in women. 2019.
4. Houben RM, Dodd PJ. The Global Burden of Latent Tuberculosis Infection: A Re-estimation Using Mathematical Modelling. PLoS Med. 2016;13(10):e1002152.
5. Organisation TWH. End TB Strategy. 2015.
6. Mathad JS, Gupta A. Tuberculosis in pregnant and postpartum women: epidemiology, management, and research gaps. Clin Infect Dis. 2012;55(11):1532-49.
7. Zenner D, Kruijshaar ME, Andrews N, Abubakar I. Risk of tuberculosis in pregnancy: a national, primary care-based cohort and self-controlled case series study. Am J Respir Crit Care Med. 2012;185(7):779-84.
8. Marais BJ, Walker TM, Cirillo DM, Raviglione M, Abubakar I, van der Werf MJ, et al. Aiming for zero tuberculosis transmission in low-burden countries. Lancet Respir Med. 2017;5(11):846-8.
9. Alsdurf H, Hill PC, Matteelli A, Getahun H, Menzies D. The cascade of care in diagnosis and treatment of latent tuberculosis infection: a systematic review and meta-analysis. Lancet Infect Dis. 2016;16(11):1269-78.
10. Kane M, Alter M, Esteban R, Rizzetto M. Role of screening in prevention and treatment. Gut. 1993;34(2 Suppl):S45-7.
11. Llewelyn M, Cropley I, Wilkinson RJ, Davidson RN. Tuberculosis diagnosed during pregnancy: a prospective study from London. Thorax. 2000;55(2):129-32.
12. Asuquo B, Vellore AD, Walters G, Manney S, Mignini L, Kunst H. A case-control study of the risk of adverse perinatal outcomes due to tuberculosis during pregnancy. J Obstet Gynaecol. 2012;32(7):635-8.
13. Zenner D. Crisis-Affected Populations and Tuberculosis. Microbiol Spectr. 2017;5(1).
14. Sobhy S, Babiker Z, Zamora J, Khan KS, Kunst H. Maternal and perinatal mortality and morbidity associated with tuberculosis during pregnancy and the postpartum period: a systematic review and meta-analysis. Bjog. 2017;124(5):727-33.
15. Tuberculosis in England: Annual Report 2019. Public Health England. 2020.
16. Abubakar I, Thomas HL, Morgan M, Anderson S, Zenner D, Cosford P. A collaborative strategy to tackle tuberculosis in England. Lancet. 2015;385(9965):312-3.

17. England PH. Collaborative Tuberculosis Strategy for England 2015-2020.
18. Zenner D, Loutet MG, Harris R, Wilson S, Ormerod LP. Evaluating 17 years of latent tuberculosis infection screening in north-west England: a retrospective cohort study of reactivation. Eur Respir J. 2017;50(1).
19. Montoy JC, Dow WH, Kaplan BC. Patient choice in opt-in, active choice, and opt-out HIV screening: randomized clinical trial. Bmj. 2016;532:h6895.
20. Young SD, Monin B, Owens D. Opt-out testing for stigmatized diseases: a social psychological approach to understanding the potential effect of recommendations for routine HIV testing. Health Psychol. 2009;28(6):675-81.
21. Wu S, Roychowdhury I, Khan M. Evaluating the impact of healthcare provider training to improve tuberculosis management: a systematic review of methods and outcome indicators used. Int J Infect Dis. 2017;56:105-10.
22. Loutet MG, Burman M, Jayasekera N, Trathen D, Dart S, Kunst H, Zenner D. National Roll-out of Latent Tuberculosis Testing and Treatment for New Migrants in England: a retrospective evaluation in a high-incidence area. Eur Respir J acceped for publication. 2017.
23. Miele K, Bamrah Morris S, Tepper NK. Tuberculosis in Pregnancy. Obstet Gynecol. 2020;135(6):1444-53.
24. Nguyen HT, Pandolfini C, Chiodini P, Bonati M. Tuberculosis care for pregnant women: a systematic review. BMC Infect Dis. 2014;14:617.
25. Merchant RC, Waxman MJ. HIV screening in health care settings: some progress, even more questions. Jama. 2010;304(3):348-9.
26. Griffiths C, Sturdy P, Brewin P, Bothamley G, Eldridge S, Martineau A, et al. Educational outreach to promote screening for tuberculosis in primary care: a cluster randomised controlled trial. Lancet. 2007;369(9572):1528-34.
27. Leber W, McMullen H, Anderson J, Marlin N, Santos AC, Bremner S, et al. Promotion of rapid testing for HIV in primary care (RHIVA2): a cluster-randomised controlled trial. Lancet HIV. 2015;2(6):e229-35.
28. Chan AW, Tetzlaff JM, Gøtzsche PC, Altman DG, Mann H, Berlin JA, et al. SPIRIT 2013 explanation and elaboration: guidance for protocols of clinical trials. Bmj. 2013;346:e7586.

Figure 1. Timeline of study project (Assessment and follow-up of migrant women)

Word count: 3909

For peer review only

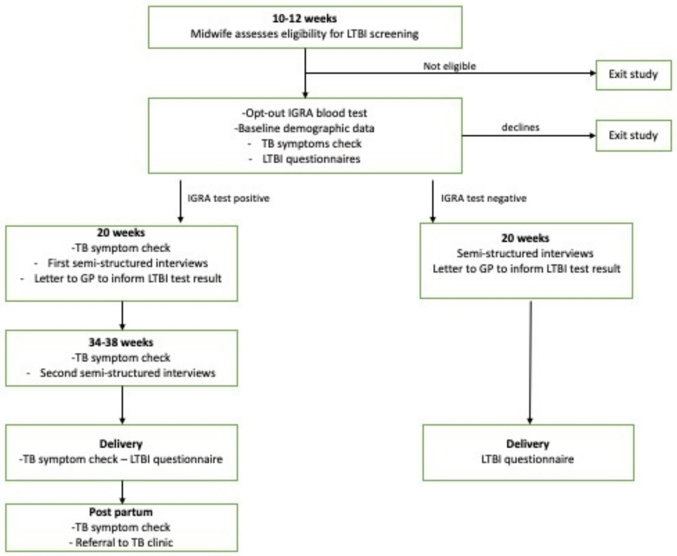

Figure 1. Timeline of study project (Assessment and follow-up of migrant women)

338x190mm (54 x 54 DPI)

Reporting checklist for protocol of a clinical trial.

Based on the SPIRIT guidelines.

Instructions to authors

Complete this checklist by entering the page numbers from your manuscript where readers will find each of the items listed below.

Your article may not currently address all the items on the checklist. Please modify your text to include the missing information. If you are certain that an item does not apply, please write "n/a" and provide a short explanation.

Upload your completed checklist as an extra file when you submit to a journal.

In your methods section, say that you used the SPIRITreporting guidelines, and cite them as:

Chan A-W, Tetzlaff JM, Gøtzsche PC, Altman DG, Mann H, Berlin J, Dickersin K, Hróbjartsson A, Schulz KF, Parulekar WR, Krleža-Jerić K, Laupacis A, Moher D. SPIRIT 2013 Explanation and Elaboration: Guidance for protocols of clinical trials. BMJ. 2013;346:e7586

		Reporting Item	Page Number
**Administrative information**			
Title	1	Descriptive title identifying the study design, population, interventions, and, if applicable, trial acronym	
Trial registration	2	Trial identifier and registry name. If not yet registered, name of intended registry	
Trial registration: data set	NA	All items from the World Health Organization Trial Registration Data Set	
Protocol version	NA	Date and version identifier	
Funding	9	Sources and types of financial, material, and other support	
Roles and responsibilities: contributorship	11	Names, affiliations, and roles of protocol contributors	

For peer review only

Roles and responsibilities: sponsor contact information	NA	Name and contact information for the trial sponsor
Roles and responsibilities: sponsor and funder	NA	Role of study sponsor and funders, if any, in study design; collection, management, analysis, and interpretation of data; writing of the report; and the decision to submit the report for publication, including whether they will have ultimate authority over any of these activities
Roles and responsibilities: committees	NA	Composition, roles, and responsibilities of the coordinating centre, steering committee, endpoint adjudication committee, data management team, and other individuals or groups overseeing the trial, if applicable (see Item 21a for data monitoring committee)
**Introduction**		
Background and rationale	3-5	Description of research question and justification for undertaking the trial, including summary of relevant studies (published and unpublished) examining benefits and harms for each intervention
Background and rationale: choice of comparators	NA	Explanation for choice of comparators
Objectives	4-5	Specific objectives or hypotheses
Trial design	5-6	Description of trial design including type of trial (eg, parallel group, crossover, factorial, single group), allocation ratio, and framework (eg, superiority, equivalence, non-inferiority, exploratory)
**Methods: Participants, interventions, and outcomes**		
Study setting	5-6	Description of study settings (eg, community clinic, academic hospital) and list of countries where data will be collected. Reference to where list of study sites can be obtained

For peer review only

Eligibility criteria	5-6	Inclusion and exclusion criteria for participants. If applicable, eligibility criteria for study centres and individuals who will perform the interventions (eg, surgeons, psychotherapists)
Interventions: description	5-6	Interventions for each group with sufficient detail to allow replication, including how and when they will be administered
Interventions: modifications	NA	Criteria for discontinuing or modifying allocated interventions for a given trial participant (eg, drug dose change in response to harms, participant request, or improving / worsening disease)
Interventions: adherance	NA	Strategies to improve adherence to intervention protocols, and any procedures for monitoring adherence (eg, drug tablet return; laboratory tests)
Interventions: concomitant care	NA	Relevant concomitant care and interventions that are permitted or prohibited during the trial
Outcomes	6	Primary, secondary, and other outcomes, including the specific measurement variable (eg, systolic blood pressure), analysis metric (eg, change from baseline, final value, time to event), method of aggregation (eg, median, proportion), and time point for each outcome. Explanation of the clinical relevance of chosen efficacy and harm outcomes is strongly recommended
Participant timeline	Figure 1	Time schedule of enrolment, interventions (including any run-ins and washouts), assessments, and visits for participants. A schematic diagram is highly recommended (see Figure)
Sample size	7	Estimated number of participants needed to achieve study objectives and how it was determined, including clinical and statistical assumptions supporting any sample size calculations
Recruitment	5-6	Strategies for achieving adequate participant enrolment to reach target sample size
**Methods: Assignment of interventions (for controlled trials)**		
Allocation: sequence generation	NA	Method of generating the allocation sequence (eg, computer-generated random numbers), and list of any factors for stratification. To reduce predictability of a random sequence, details of any planned restriction (eg, blocking) should be

For peer review only

		provided in a separate document that is unavailable to those who enrol participants or assign interventions
Allocation concealment mechanism	NA	Mechanism of implementing the allocation sequence (eg, central telephone; sequentially numbered, opaque, sealed envelopes), describing any steps to conceal the sequence until interventions are assigned
Allocation: implementation	NA	Who will generate the allocation sequence, who will enrol participants, and who will assign participants to interventions
Blinding (masking)	NA	Who will be blinded after assignment to interventions (eg, trial participants, care providers, outcome assessors, data analysts), and how
Blinding (masking): emergency unblinding	NA	If blinded, circumstances under which unblinding is permissible, and procedure for revealing a participant's allocated intervention during the trial
**Methods: Data collection, management, and analysis**		
Data collection plan	8-9	Plans for assessment and collection of outcome, baseline, and other trial data, including any related processes to promote data quality (eg, duplicate measurements, training of assessors) and a description of study instruments (eg, questionnaires, laboratory tests) along with their reliability and validity, if known. Reference to where data collection forms can be found, if not in the protocol
Data collection plan: retention	8-9	Plans to promote participant retention and complete follow-up, including list of any outcome data to be collected for participants who discontinue or deviate from intervention protocols
Data management	8-9	Plans for data entry, coding, security, and storage, including any related processes to promote data quality (eg, double data entry; range checks for data values). Reference to where details of data management procedures can be found, if not in the protocol

For peer review only

Statistics: outcomes	7	Statistical methods for analysing primary and secondary outcomes. Reference to where other details of the statistical analysis plan can be found, if not in the protocol
Statistics: additional analyses	7	Methods for any additional analyses (eg, subgroup and adjusted analyses)
Statistics: analysis population and missing data	NA	Definition of analysis population relating to protocol non-adherence (eg, as randomised analysis), and any statistical methods to handle missing data (eg, multiple imputation)
**Methods: Monitoring**		
Data monitoring: formal committee	NA	Composition of data monitoring committee (DMC); summary of its role and reporting structure; statement of whether it is independent from the sponsor and competing interests; and reference to where further details about its charter can be found, if not in the protocol. Alternatively, an explanation of why a DMC is not needed
Data monitoring: interim analysis	NA	Description of any interim analyses and stopping guidelines, including who will have access to these interim results and make the final decision to terminate the trial
Harms	NA	Plans for collecting, assessing, reporting, and managing solicited and spontaneously reported adverse events and other unintended effects of trial interventions or trial conduct
Auditing	NA	Frequency and procedures for auditing trial conduct, if any, and whether the process will be independent from investigators and the sponsor
**Ethics and dissemination**		
Research ethics approval	9	Plans for seeking research ethics committee / institutional review board (REC / IRB) approval
Protocol amendments	NA	Plans for communicating important protocol modifications (eg, changes to eligibility criteria, outcomes, analyses) to relevant parties (eg, investigators, REC / IRBs, trial participants, trial registries, journals, regulators)

For peer review only

Consent or assent	NA	Who will obtain informed consent or assent from potential trial participants or authorised surrogates, and how (see Item 32)
Consent or assent: ancillary studies	NA	Additional consent provisions for collection and use of participant data and biological specimens in ancillary studies, if applicable
Confidentiality	8-9	How personal information about potential and enrolled participants will be collected, shared, and maintained in order to protect confidentiality before, during, and after the trial
Declaration of interests	11	Financial and other competing interests for principal investigators for the overall trial and each study site
Data access	8-9	Statement of who will have access to the final trial dataset, and disclosure of contractual agreements that limit such access for investigators
Ancillary and post trial care	NA	Provisions, if any, for ancillary and post-trial care, and for compensation to those who suffer harm from trial participation
Dissemination policy: trial results	9	Plans for investigators and sponsor to communicate trial results to participants, healthcare professionals, the public, and other relevant groups (eg, via publication, reporting in results databases, or other data sharing arrangements), including any publication restrictions
Dissemination policy: authorship	NA	Authorship eligibility guidelines and any intended use of professional writers
Dissemination policy: reproducible research	NA	Plans, if any, for granting public access to the full protocol, participant-level dataset, and statistical code
**Appendices**		
Informed consent materials	NA	Model consent form and other related documentation given to participants and authorised surrogates
Biological specimens	NA	Plans for collection, laboratory evaluation, and storage of biological specimens for genetic or molecular analysis in the current trial and for future use in ancillary studies, if applicable

None The SPIRIT Explanation and Elaboration paper is distributed under the terms of the Creative Commons Attribution License CC-BY-NC. This checklist can be completed online using https://www.goodreports.org/, a tool made by the EQUATOR Network in collaboration with Penelope.ai

For peer review only
